# SHAP analysis of an improved EEG-based mental workload classification framework: utilizing data augmentation and explainable AI

**DOI:** 10.1038/s41598-026-52330-z

**Published:** 2026-05-20

**Authors:** Sushil Chaturvedi, Mitul Kumar Ahirwal

**Affiliations:** Department of Computer Science and Engineering, Maulana Azad National Institute of Technology, Bhopal, M.P. India

**Keywords:** Electroencephalography (EEG), Mental workload (MWL), EEGNet, Synthetic minority oversampling technique (SMOTE), Explainable artificial intelligence (XAI), Computational biology and bioinformatics, Engineering, Mathematics and computing, Neuroscience

## Abstract

Mental workload (MWL) classification using electroencephalogram (EEG) signals is crucial for cognitive neuroscience and is also a challenging research area in brain-computer interface (BCI). Since the EEG signals fluctuate a lot across sessions and individuals, there is a need for a robust classification model that generalizes well for real-world applications. In this work, we used the publicly available dataset “An EEG dataset for cross-session mental workload estimation: passive BCI competition of the Neuroergonomics Conference 2021”, and the standard EEGNet model to classify the MWL into three classes (Low, Med, and High). To improve the performance of the model, a synthetic minority oversampling technique (SMOTE) was used by creating synthetic EEG samples, and key hyperparameters (*F*_1_, *F*_2_, and *D*) of EEGNet were systematically varied to identify the optimal configuration. Furthermore, Shapley Additive Explanations (SHAP) analysis was performed to identify the most influential EEG channels for model prediction. The proposed approach achieves the highest accuracy of 80.5% and 82.7% without and with SMOTE, respectively. The comparative analysis showed that applying SMOTE resulted in an average performance improvement of approximately 3%. A Wilcoxon signed-rank test confirmed that this improvement was statistically significant (*p* < 0.05). Finally, the SHAP analysis revealed that the most informative EEG channels were located over the parieto-occipital and temporal regions, which is consistent with established neurophysiological evidence related to MWL processing. The proposed framework improves both performance and explainability in EEG-based MWL classification, representing a systematic integration of SMOTE and SHAP analysis.

## Introduction

Mental workload (MWL) classification through electroencephalogram (EEG) signals is an important field of study that has various applications in cognitive neuroscience, human–computer interaction (HCI), and brain-computer interface (BCI). Advancements in automation reduce the human operating load, but sometimes it also increases the human’s mental workload. A high mental workload can cause severe trouble in human–computer interactive systems, like anxiety^1^, stress^2^, tiredness^3^, etc., resulting in degradation of the overall performance of the system. It is possible to monitor real-time cognitive states by classifying MWL through EEG signals, which can improve performance across a range of sectors, including education, healthcare, driving, and aviation.

Mental workload assessment can be performed in two ways: (1) subjective assessment, and (2) physiological assessment^4,5^. In subjective assessment techniques like the NASA Task Load Index (NASA-TLX) and Subjective Workload Assessment Technique (SWAT), the participants are asked to rate their workload in the form of questionnaires after completion of the task. The subjective assessment does not give accurate results compared to the physiological assessment. Subjective assessment depends on personal perception that can be biased by way of expression, limitations of memory, emotion at the time of rating, feelings, and the social interest of the subject. Conversely, physiological assessments that can be conducted using EEG^6^, heart rate variability (HRV), electrocardiogram (ECG)^7^, and other methods provide direct real-time responses from the brain or heart, thereby avoiding self-response biases. EEG has become the most widely used technique^8–10^ among such techniques because it is non-invasive, easy to carry, and relatively low-cost.

To better understand the current progress in EEG-based MWL assessment, it is essential to review the existing state-of-the-art research and examine the methodologies and findings reported in previous studies. Chaturvedi S et al.^11^ have examined different validation and testing methods to observe the influence of the performance of MWL classification across multiple recording sessions. They also explored transfer-learning techniques to reduce cross-session variability and performed SHAP analysis to explain the model’s predictions and to determine the EEG features that contributed most. To capture the spatial, spectral, and temporal features together, Guan et al.^12^ proposed a cross-task MWL assessment method that represents EEG signals in tensor form. They have also used transfer learning to transfer knowledge between different tasks, improving generalization when training data for a new task is limited. Beiramvand et al.^13^ proposed a MWL classification technique using a transformer network with only two prefrontal EEG channels, making the framework that is suitable for wearable devices. They have used parameter-free entropy and energy features and achieved consistent performance across different EEG devices while reducing the model complexity. Kongwudhikunakorn et al.^14^ introduced EEGMeNet, an end-to-end multitask neural network that together learns MWL levels and related brain features directly from EEG signals. By sharing representations across tasks, the model improves classification accuracy and reduces the need for handcrafted EEG features. Guan et al.^15^ developed a multi-branch LSTM model using an attention mechanism to evaluate the MWL of crew members during tunnel navigation tasks. They have combined the information from multiple temporal branches and focusing on important EEG features, improved the model recognition performance in complex and dynamic navigation environments. Sharma et al.^16^ proposed a deep learning-based technique for the assessment of MWL using the EEG signals. They processed the EEG signals with a discrete wavelet transform and Welch’s power spectral density. Combination of time–frequency EEG features with deep learning models, the method improves MWL classification accuracy compared to traditional feature-based techniques. EEG signals have essential time–frequency information that can be effectively analyzed by decomposing them into different bands using the wavelet transform. Hassan J et al.^17^ proposed a framework for MWL estimation that used the wavelet transform for multi-resolution signal decomposition and statistical feature extraction. The extracted features are then classified using a neural network classifier, demonstrating that wavelet-based time–frequency features can effectively discriminate different workload levels. Recently, some attention-based and large-scale pretrained models have been introduced to improve EEG representation learning and decoding performance. Aziz et al.^18^ proposed BCINetV1, a convolutional attention-based framework that focuses on important temporal and frequency patterns in motor imagery EEG signals. Jiang et al.^19^ have proposed LaBraM, a large pretrained transformer-based model that learns universal EEG representations from large multi-dataset data using masked EEG modeling and vector-quantized spectrum prediction. Wang et al.^20^ introduced EEGPT, a pretrained transformer-based framework that learns universal EEG features by using masked reconstruction and spatio-temporal alignment, which improves the performance across different BCI tasks.

According to the literature mentioned above, the majority of studies have used EEG data that is recorded in a single session. Because the EEG signals are not stationary, they may undergo substantial changes over days or even within a single day for the same person. This variability can affect the generalizability of machine learning or deep learning models trained on a single session dataset, as they can provide poor results when they are applied to data that has been recorded in multiple sessions or used in real-time BCI systems. To address these challenges, in this work, a dataset was used that has been recorded in multiple sessions, which allows us to deal with multiple-session variability and improve the robustness and generalizability of the classification model. Here, the classification of the EEG data has been done using the EEGNet model, and to improve the performance of the model, a synthetic minority oversampling technique (SMOTE) and tuning of key parameters (*F*_1_,* F*_2_, and* D*) of the EEGNet model were applied. Along with this, the SHAP (Shapley Additive explanation), an explainable artificial intelligence (XAI) approach, is used to determine which features are important for MWL prediction.

To the best of our knowledge, this is the first study that jointly uses EEGNet, SMOTE, and SHAP for MWL assessment, addressing both the EEG data scarcity limitation and model interpretability within a single unified framework. We also investigated the influence of the EEGNet network parameters (*F*1, *F*2, and *D*) by varying their values and showed continuous performance gains. Through SHAP analysis, we provided interpretability and determined which EEG channels are more influential in the model prediction.

The main contributions of this work are as follows:

1. Evaluating EEGNet performance by varying its parameters (*F*_1_,* F*_2_, and* D*) using the cross-session mental workload dataset.
2. Examined the effect of SMOTE-based synthetic sample generation on model performance, demonstrating that EEGNet’s accuracy and stability are enhanced by more training examples in a variety of hyperparameter combinations *(F*_1_, *F*_2_, and *D*).
3. Applied SHAP analysis to interpret the EEGNet prediction, identifying the most influential EEG channels for MWL classification.

The remainder of the paper is organized as follows: the methodology is presented in section “Methodology”, the Results are presented in section “Results”, the discussion is presented in section “Discussion”, followed by the conclusion.

## Methodology

This study investigates EEG-based MWL classification over a cross-session dataset to better reflect real-world situations. The strategy uses the XAI technique to identify the most pertinent channels for model prediction and the SMOTE technique to enhance the EEGNet model’s performance. Further in this section, the details of the dataset, the EEGNet model, SMOTE, performance parameters, and XAI techniques are described.

### Datasets

In this work, an EEG dataset for cross-session mental workload estimation: passive BCI competition of the Neuroergonomics Conference 2021^21^ was used; an overview of this dataset is given below:

Cross-session mental workload dataset:

In this dataset, a total of 15 participants (9 male and 6 female) performed the multi-attribute task battery–II (MATB-II) task in three sessions separated by one week; the task consisted of three levels of difficulty (low, medium, and high) per session. This dataset is open source and can be found at https://zenodo.org/records/5055046.

**Fig. 1:**
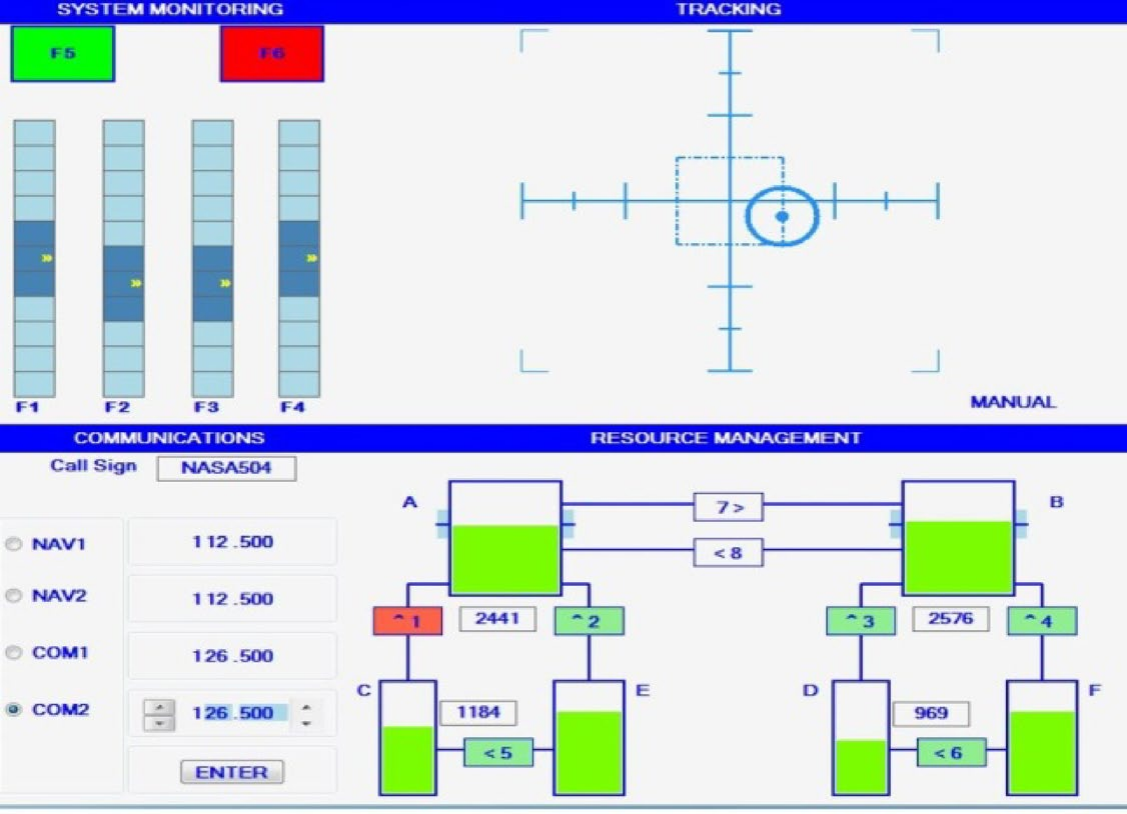
Screenshot of MATB-II with four subtasks: System monitoring, tracking, communication, and fuel management^19^.

**Fig. 2:**
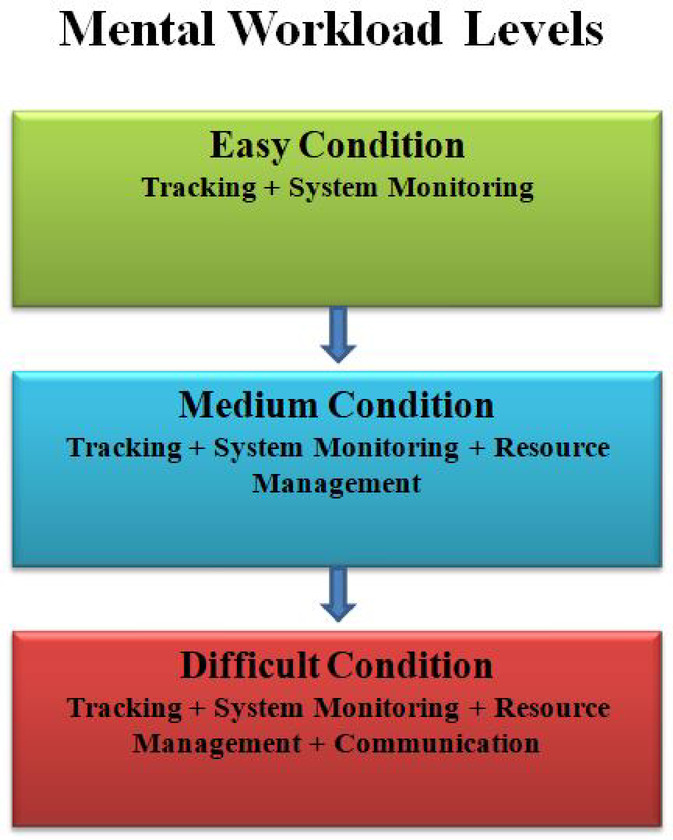
Different levels of MWL.

- *Data acquisition*: The EEG data is recorded using a 64 Ag–AgCl electrode system placed in an international 10–20 arrangement system. One electrode is used to record the cardiac activity, and one electrode cannot be used, so a total of 62 electrode data were used. A sampling frequency is set to 500 Hz, and impedance is set to below 10 kΩ as much as possible.
- *Task*: To record the EEG data of various levels of mental workload, participants have performed a well-known MATB-II task^22^ developed by NASA. In MATB-II, there are different numbers of subtasks and varying levels of complexity depending on the condition that the participants must complete concurrently. For easy conditions, the participant has to perform two tasks concurrently: one is the tracking, and the other is the system monitoring sub-task. The tracking task requires the participant to maintain the target at the center of the display. For medium conditions, a third sub-task, resource management, is added, where the objective is to keep a fuel tank’s fuel level at a specific level. Finally, for difficult conditions, the communication sub-task was also included in prior sub-tasks, where the participant had to reply to the radio messages by tuning the frequencies of various radios. The screenshot of the MATB-II task is shown in Fig. 1, and how the mental workload changes from easy to difficult conditions is shown in Fig. 2.
- *Data preprocessing*: The recorded EEG data were preprocessed in MATLAB with the help of the EEGLAB toolbox^23^. The following steps are taken for preprocessing:
  - The data were epoched into non-overlapping 2-s segments
  - Referencing was performed using the right mastoid electrode
  - The EEG signals were high-pass filtered at 1 Hz to eliminate slow baseline drifts and movement artifacts.
  - A low-pass filter with a frequency of 40 Hz was used to reduce muscle activity noise and other unwanted high-frequency signals.
  - Initially, the EEG signals were captured at 500 Hz and were later reduced to 250 Hz to reduce computational load while preserving the important information.

### EEGNet model

**Fig. 3:**
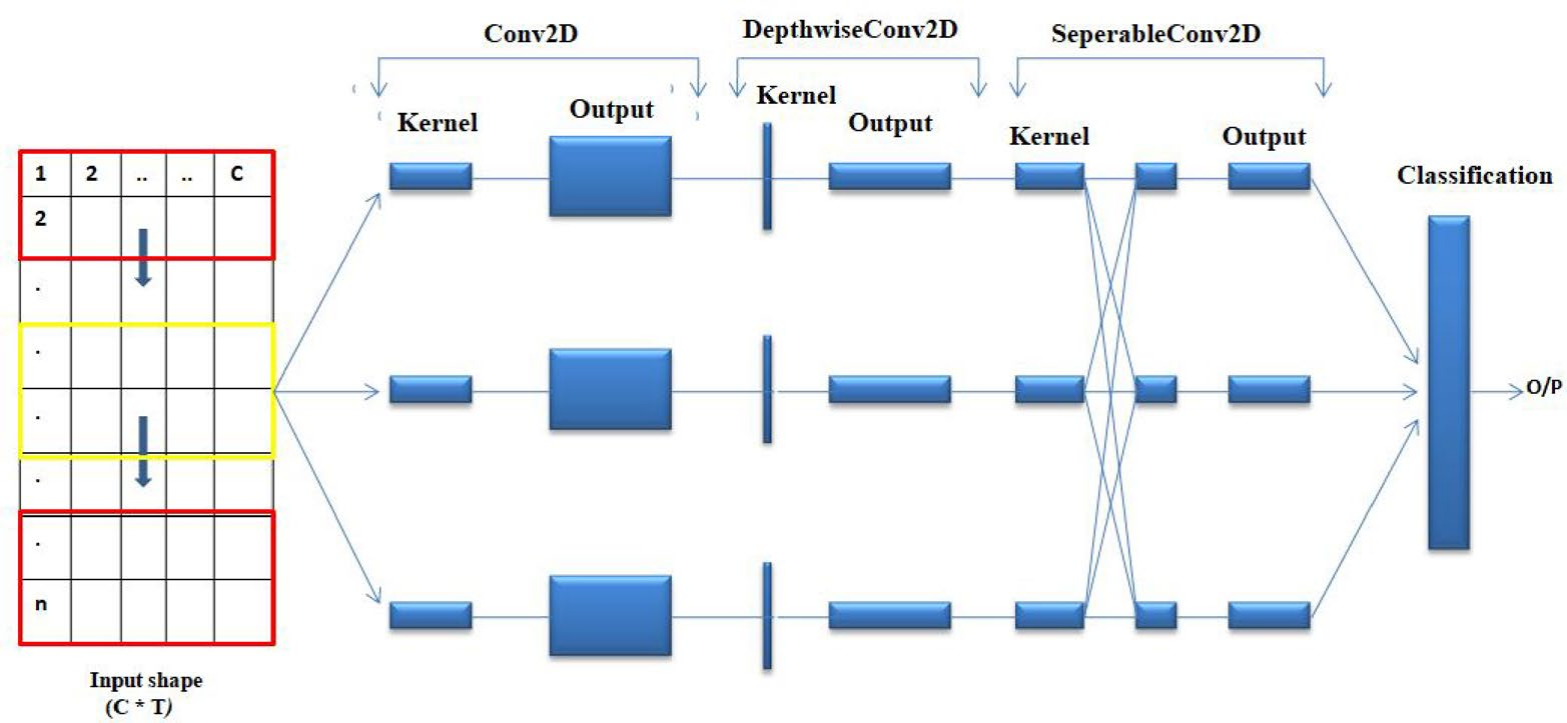
Block diagram of EEGNet. The input is (*C* × *T*, with *C* denoting the number of channels and *T* is time points), Line represents the connectivity, block1 represents temporal convolution, block 2 represents depthwise convolution, block 3 represents separable convolution, and lastly the classification layer.

EEGNet is a lightweight and computationally efficient deep learning model that is specially designed for EEG-based brain-computer interface (BCI) applications^24^. EEGNet has various capabilities; it can be used for a variety of BCI scenarios (like mental workload classification, motor imagery task, and event-related potential, etc.), and can provide features that can be understood by neurophysiology. A block diagram of EEGNet is shown in Fig. 3. As seen from the block diagram, first, the input (*C* × *T*) is given to the *F*_1_ filter of size (1, 64) in our case, which produces *F*_1_ feature maps, and after this, a depthwise convolution is used of size (*C*, 1), where *C* is the number of channels. The primary advantage of using depthwise convolution is that it decreases the number of trainable parameters because these convolutions aren’t entirely coupled to all of the earlier feature maps. A depth parameter *D* decides how many spatial filters will be used for each temporal filter (in Fig. 3, *D* = 1 is shown for simplicity). The output of a depthwise convolution is then passed to a separable convolution; a separable convolution consists of a depthwise convolution of size (1, 16) in our case, followed by a pointwise convolution *F*_2_ of size (1, 1).

The two primary advantages of separable convolutions are (1) lowering the number of parameters and (2) explicitly decoupling the relationship between and within feature maps by first learning a kernel that summarizes each feature map separately, followed by merging the outputs. The output of separable convolution is then passed to the classification layer that consists of *N* units, where *N* is the number of classes in the data. Along the feature map dimension, a batch normalization layer, an exponential linear unit (ELU) to add nonlinearity, followed by a dropout layer, which is set to 0.5, was also used. The model summary is shown in Table 1 for the configuration: *F*_1_ = 4, *F*_2_ = 8, and *D* = 2.

**Table 1:** EEGNet model summary for *F*_1_ = 4, *F*_2_ = 8, and *D* = 2 configuration.

Type of layer	Output shape	Parameters
Input layer	(None, 61, 500, 1)	0
Conv2D	(None, 61, 500, 4)	256
Batch Normalization layer	(None, 61, 500, 4)	16
DepthwiseConv2D	(None, 1, 500, 8)	488
Batch Normalization layer	(None, 1, 500, 8)	32
Activation	(None, 1, 500, 8)	0
Average Pooling2D	(None, 1, 125, 8)	0
Dropout	(None, 1, 125, 8)	0
SeparableConv2D	(None, 1, 125, 8)	192
Batch Normalization layer	(None, 1, 125, 8)	32
Activation	(None, 1, 125, 8)	0
AveragePooling2D	(None, 1, 15, 8)	0
Dropout	(None, 1, 15, 8)	0
Flatten	(None, 120)	0
Dense layer	(None, 3)	363
Activation (SoftMax)	(None, 3)	0
Total parameters: 1379 (5.39 KB) Trainable parameters: 1339 (5.23 KB) Non-trainable parameters: 40 (160.00 Byte)		

### SMOTE

SMOTE is an oversampling technique that generates synthetic samples using interpolation between existing samples instead of oversampling using replacement^25^. This technique is motivated by a method that was used in handwritten character detection^26^ to generate additional training data; they applied specific techniques like rotation and skew to actual data. In SMOTE, the oversampling is performed by taking each minority class sample and adding synthetic samples along the line segments joining any or all of the *k*-minority class nearest neighbors. Neighbors from the *k* nearest neighbors are selected at random, depending on how much oversampling is needed. In this work, SMOTE was applied at the epoch level on normalized EEG data (only the training set). Each EEG trial was treated as an independent fixed-length epoch, which was flattened into a feature vector only for the interpolation step and then reshaped back to its original structure before being given to the model^27,28^. Since the interpolation occurs between samples belonging to the same class and identical epoch length, the within-epoch temporal ordering was preserved. The test set was not touched in SMOTE to avoid data leakage. The following algorithm describes the implementation of SMOTE:

1. Flattening the EEG data:
  Our data has shape (samples, channels, time points).
  Reshape the data into (samples, channels × time points).
2. Finding the nearest neighbors:
  For each class sample*Xi,* SMOTE uses *k* nearest neighbors (in this case, *k* = 5) using the Euclidean distance.
3. Synthetic sample generation:
  A random neighbor *Xn* was selected.
  A synthetic sample *Xnew* was created using linear interpolation.
  $Xnew = Xi + \lambda \times (Xn - Xi),$ where λ is a random number in the range of 0 to 1.
4. Reshaping the data back to its original shape.

### Performance parameters

Performance indicators for the EEGNet model include classification accuracy (ACC), precision (PRC), recall (RCL), and F1-score. The calculation of the accuracy, precision, recall, and F1-score is determined using Eqs. (1)–(4), respectively.

1 $${\mathrm{ACC}} = \frac{{{\text{TP of class j}}}}{{{\text{Total number of instances of all the classes}}}},$$

2 $${\text{PRC }} = \frac{{{\text{TP of class j}}}}{{{\text{Total number of classes predicted as class j }}}},$$

3 $${\text{RCL }} = \frac{{{\text{TP of class j}}}}{{{\text{Number of instances of class j}}}},$$

4 $${\mathrm{F}}1{\mathrm{-Score}} = 2*\frac{{{\mathrm{PCR*RCL}}}}{{{\mathrm{PCR}} + {\mathrm{RCL}}}}$$

### Explainable AI (XAI)

XAI is a group of methods aimed at interpreting deep learning models and making them more transparent and reliable. Because deep learning models are more complicated and frequently referred to as “black box” models, it is vital to comprehend how and why they produce predictions to comprehend their decision-making process^29^. SHAP is the most popular XAI technique for interpreting models and making explanations.

#### SHAP (Shapley additive explanation)

A well-known technique for analyzing machine learning (ML) or deep learning (DL) model predictions is Shapley Additive Explanation (SHAP). The SHAP theory was first coined by Lundberg and Lee^30^. The calculation of SHAP values is based on game theory^31^ to disclose how each attribute contributes to particular predictions. SHAP is a model-agnostic technique that can explain any variety of models (machine learning or deep learning models). Since the DL models are a kind of black box, by giving individual input qualities significance, this method helps us to understand the behavior of the DL model. Mathematically, Shapley values are represented by the following equation-

5 $${\phi}_{i}=\sum_{S\subseteq N\backslash \{i\}}\frac{\left|S\right|!\left(\left|N\right|-\left|S\right|-1\right)!}{\left|N\right|!} [f\left(S\cup \left\{i\right\}-f\left(S\right)\right]$$

Here, *Φ*_*i*_ is the Shapley value for feature *i*, *N* is the set of all features, *S* is the subset of features excluding *i*, *f*(*S*) is the model output with subset* S*, *f*(S$\cup${*i*}) is the model output when adding *I* to subset, and |S!(|N|-|S|-1)!/|N!| This term ensures the marginal contribution of feature *i* is averaged across all potential feature subsets.

The formula calculates the average marginal contribution of feature *i* across all possible feature subsets. It ensures that features are properly attributed according to how they affect the model’s output. Because of this, SHAP values can be used with any ML or DL model, making them model-agnostic.

## Results

This section discusses the classification performance results of the aforementioned methodologies. EEGNet has parameters such as an *F*1 temporal filter, an *F*2 point-wise filter, and a depth multiplier *D*. The experiments were performed by varying these parameters in the following manner, as shown in Table 2.

**Table 2:** Various configurations by varying the parameters (*F*_1_, *F*_2_, and *D*) of the EEGNet.

Serial No	*D* = 2	*D* = 4
1	*F*_1_ = 4, *F*_2_ = 8	*F*_1_ = 4, *F*_2_ = 8
2	*F*_1_ = 8, *F*_2_ = 16	*F*_1_ = 8, *F*_2_ = 16
3	*F*_1_ = 16, *F*_2_ = 32	*F*_1_ = 16, *F*_2_ = 32
4	*F*_1_ = 32, *F*_2_ = 64	*F*_1_ = 32, *F*_2_ = 64
5	*F*_1_ = 64, *F*_2_ = 128	*F*_1_ = 64, *F*_2_ = 128

**Fig. 4:**
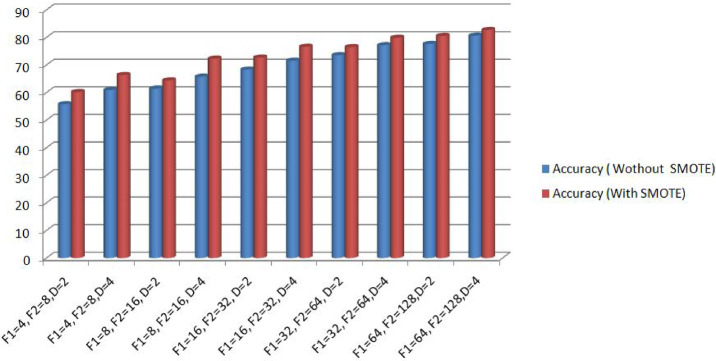
Bar chart showing the accuracy of all configurations.

In this way, a total of 20 experiments, 10 without SMOTE and 10 with SMOTE, are computed. To improve the performance, the SMOTE technique was used, which increases the number of samples. The model’s effect on classification accuracy was investigated by training and assessing it in two scenarios: one with SMOTE-augmented data and the other with the original dataset, i.e., without SMOTE, for all configurations. An Adam optimizer with categorical cross-entropy loss was used, and the SoftMax activation function was applied in the output layer to classify the data into three classes. For every configuration, the model is trained for 50 epochs and repeated across five independent trials to ensure the reliability of the results. Tables 3, 4, 5, 6, 7, 8, 9, 10, 11, 12, 13, 14, 15, 16, 17, 18, 19, 20, 21 and 22 show the results of each configuration as mentioned in Table 2; the average mean ± standard deviation of all configurations is shown in Table 23, and the bar chart of the accuracies of configurations is shown in Fig. 4.

**Table 3:** MWL classification results without SMOTE for *F*_1_ = 4,* F*_2_ = 8, and *D* = 2.

Trial	Accuracy	Precision	Recall	F-1 Score
1	55.264	53.514	55.264	50.915
2	56.129	53.733	56.129	52.637
3	55.980	53.281	55.980	51.176
4	56.517	55.415	56.517	52.441
5	54.846	52.707	54.846	50.320
Mean	55.747	53.730	55.747	51.498
Std. Dev	0.6780	1.0170	0.6780	1.0020

**Table 4:** MWL classification results with SMOTE for *F*_1_ = 4,* F*_2_ = 8, and *D* = 2.

Trial	Accuracy	Precision	Recall	F-1 Score
1	60.45	59.41	60.45	59.26
2	58.96	58.38	58.96	55.87
3	61.14	58.98	61.14	58.61
4	61.17	59.34	61.17	59.39
5	59.17	56.82	59.17	56.69
Mean	60.18	58.59	60.18	57.96
Std. Dev	1.060	1.070	1.060	1.590

**Table 5:** MWL classification results without SMOTE for *F*_1_ = 8,* F*_2_ = 16, and *D* = 2.

Trial	Accuracy	Precision	Recall	F-1 Score
1	60.781	58.797	60.781	56.031
2	60.752	59.187	60.752	56.523
3	63.883	62.611	63.883	61.318
4	60.602	58.589	60.602	56.985
5	61.169	59.23	61.169	57.285
Mean	61.438	59.683	61.438	57.628
Std. Dev	1.3830	1.6590	1.3830	2.1170

**Table 6:** MWL classification results with SMOTE for *F*_1_ = 8,* F*_2_ = 16, and *D* = 2.

Trial	Accuracy	Precision	Recall	F-1 Score
1	65.34	64.79	65.34	63.37
2	68.24	67.88	68.24	67.36
3	63.08	62.01	63.08	60.54
4	61.71	60.53	61.71	60.55
5	63.67	61.94	63.67	61.46
Mean	64.41	63.43	64.41	62.66
Std. Dev	2.510	2.930	2.510	2.870

**Table 7:** MWL classification results without SMOTE for *F*_1_ = 16,* F*_2_ = 32, and *D* = 2.

Trial	Accuracy	Precision	Recall	F-1 Score
1	68.655	68.482	68.655	66.728
2	68.625	68.057	68.625	67.041
3	68.536	67.804	68.536	67.564
4	68.237	67.783	68.237	66.383
5	67.402	67.056	67.402	65.265
Mean	68.291	67.836	68.291	66.596
Std. Dev	0.5240	0.5190	0.5240	0.8610

**Table 8:** MWL classification results with SMOTE for *F*_1_ = 16,* F*_2_ = 32, and *D* = 2.

Trial	Accuracy	Precision	Recall	F-1 Score
1	70.59	70.05	70.59	69.96
2	73.40	73.16	73.40	72.76
3	72.11	72.04	72.11	71.50
4	72.56	72.27	72.56	71.88
5	74.68	74.35	74.68	74.23
Mean	72.67	72.38	72.67	72.07
Std. Dev	1.520	1.580	1.520	1.580

**Table 9:** MWL classification results without SMOTE for *F*_1_ = 32,* F*_2_ = 64, and *D* = 2.

Trial	Accuracy	Precision	Recall	F-1 Score
1	74.799	74.745	74.799	74.003
2	72.532	72.526	72.532	71.352
3	72.353	71.808	72.353	71.559
4	75.007	74.978	75.007	74.359
5	73.069	72.719	73.069	72.230
Mean	73.552	73.355	73.552	72.701
Std. Dev	1.2630	1.4180	1.2630	1.3960

**Table 10:** MWL classification results with SMOTE for *F*_1_ = 32,* F*_2_ = 64, and *D* = 2.

Trial	Accuracy	Precision	Recall	F-1 Score
1	74.77	74.90	74.77	74.24
2	77.90	77.91	77.90	77.73
3	77.36	77.37	77.36	76.92
4	76.59	76.42	76.59	76.36
5	75.63	75.62	75.63	75.29
Mean	76.45	76.44	76.45	76.11
Std. Dev	1.270	1.230	1.270	1.370

**Table 11:** MWL classification results without SMOTE for *F*_1_ = 64,* F*_2_ = 128, and *D* = 2.

Trial	Accuracy	Precision	Recall	F-1 Score
1	77.692	77.431	77.692	77.322
2	77.393	77.232	77.393	76.969
3	77.125	77.171	77.125	76.515
4	78.199	77.968	78.199	77.866
5	77.662	77.383	77.662	77.365
Mean	77.614	77.437	77.614	77.207
Std. Dev	0.3990	0.3150	0.3990	0.5020

**Table 12:** MWL classification results with SMOTE for *F*_1_ = 64,* F*_2_ = 128, and *D* = 2.

Trial	Accuracy	Precision	Recall	F-1 Score
1	80.05	79.86	80.05	79.81
2	80.32	80.23	80.32	80.10
3	80.55	80.49	80.55	80.28
4	80.70	80.75	80.70	80.36
5	80.88	80.77	80.88	80.71
Mean	80.50	80.42	80.50	80.25
Std. Dev	0.330	0.380	0.330	0.330

**Table 13:** MWL classification results without SMOTE for *F*_1_ = 4,* F*_2_ = 8, and *D* = 4.

Trial	Accuracy	Precision	Recall	F-1 Score
1	61.199	59.166	61.199	57.180
2	60.334	59.558	60.334	57.110
3	62.213	60.735	62.213	59.161
4	61.467	60.226	61.467	57.402
5	59.290	57.256	59.290	54.784
Mean	60.901	59.388	60.901	57.127
Std. Dev	1.1230	1.3360	1.1230	1.5580

**Table 14:** MWL classification results with SMOTE for *F*_1_ = 4,* F*_2_ = 8, and *D* = 4.

Trial	Accuracy	Precision	Recall	F-1 Score
1	66.75	66.08	66.75	65.14
2	65.17	63.86	65.17	63.21
3	66.98	66.23	66.98	65.74
4	67.49	66.52	67.49	66.13
5	65.31	63.80	65.31	62.94
Mean	66.34	65.30	66.34	64.63
Std. Dev	1.040	1.350	1.040	1.470

**Table 15:** MWL classification results without SMOTE for *F*_1_ = 8,* F*_2_ = 16, and *D* = 4.

Trial	Accuracy	Precision	Recall	F-1 Score
1	66.955	66.405	66.955	65.069
2	67.611	67.404	67.611	65.414
3	64.748	63.428	64.748	62.036
4	65.613	64.590	65.613	63.024
5	63.764	62.791	63.764	61.164
Mean	65.738	64.924	65.738	63.341
Std. Dev	1.5720	1.9520	1.5720	1.8590

**Table 16:** MWL classification results with SMOTE for *F*_1_ = 8,* F*_2_ = 16, and *D* = 4.

Trial	Accuracy	Precision	Recall	F-1 Score
1	72.23	72.56	72.23	71.05
2	72.17	72.97	72.17	70.79
3	70.98	70.29	70.98	70.10
4	72.56	72.71	72.56	71.43
5	73.40	73.21	73.4	72.75
Mean	72.27	72.35	72.27	71.22
Std. Dev	0.870	1.180	0.870	0.980

**Table 17:** MWL classification results without SMOTE for *F*_1_ = 16,* F*_2_ = 32, and *D* = 4.

Trial	Test Acc	Precision	Recall	F-1 Score
1	70.922	70.558	70.922	69.736
2	72.085	71.962	72.085	70.891
3	72.025	71.433	72.025	71.103
4	71.906	71.771	71.906	70.635
5	70.802	70.362	70.802	69.612
Mean	70.922	70.558	70.922	69.736
Std. Dev	72.085	71.962	72.085	70.891

**Table 18:** MWL classification results with SMOTE for *F*_1_ = 16,* F*_2_ = 32, and *D* = 4.

Trial	Accuracy	Precision	Recall	F-1 Score
1	76.74	76.67	76.74	76.51
2	76.80	76.58	76.80	76.45
3	76.41	76.31	76.41	75.93
4	75.48	75.50	75.48	75.12
5	77.48	77.35	77.48	77.07
Mean	76.58	76.48	76.58	76.22
Std. Dev	0.730	0.670	0.730	0.730

**Table 19:** MWL classification results without SMOTE for *F*_1_ = 32,* F*_2_ = 64, and *D* = 4.

Trial	Accuracy	Precision	Recall	F-1 Score
1	76.94	76.90	76.94	76.48
2	76.17	75.98	76.17	75.57
3	77.75	77.56	77.75	77.29
4	77.06	76.89	77.06	76.64
5	77.93	77.70	77.93	77.48
Mean	77.17	77.01	77.17	76.69
Std. Dev	0.703	0.685	0.703	0.753

**Table 20:** MWL classification results with SMOTE for *F*_1_ = 32,* F*_2_ = 64, and *D* = 4.

Trial	Accuracy	Precision	Recall	F-1 Score
1	80.58	80.51	80.58	80.31
2	79.57	79.62	79.57	79.19
3	79.93	79.75	79.93	79.69
4	79.72	79.63	79.72	79.46
5	79.54	79.49	79.54	79.13
Mean	79.87	79.80	79.87	79.56
Std. Dev	0.430	0.410	0.430	0.480

**Table 21:** MWL classification results without SMOTE for *F*_1_ = 64,* F*_2_ = 128, and *D* = 4.

Trial	Accuracy	Precision	Recall	F-1 Score
1	81.420	81.290	81.420	81.276
2	81.151	81.001	81.151	80.935
3	79.571	79.478	79.571	79.219
4	80.406	80.232	80.406	80.192
5	80.376	80.215	80.376	80.182
Mean	80.585	80.443	80.585	80.361
Std. Dev	0.7280	0.7170	0.7280	0.7960

**Table 22:** MWL classification results with SMOTE for *F*_1_ = 64,* F*_2_ = 128, and *D* = 4.

Trial	Accuracy	Precision	Recall	F-1 Score
1	82.37	82.45	82.37	82.16
2	83.66	83.79	83.66	83.40
3	82.11	82.01	82.11	81.93
4	82.94	82.87	82.94	82.78
5	82.22	82.20	82.22	81.99
Mean	82.66	82.67	82.66	82.45
Std. Dev	0.640	0.710	0.640	0.630

**Table 23:** Overall classification accuracy (%) of all configurations, reported as mean ± standard deviation.

Parameters Setting	Without SMOTE (Accuracy %)	With SMOTE (Accuracy %)
*F*_1_ = 4, *F*_2_ = 8, and *D* = 2	55.747 ± 0.678	60.18 ± 1.06
*F*_1_ = 4, *F*_2_ = 8, and *D* = 4	60.901 ± 1.123	66.34 ± 1.04
*F*_1_ = 8*, F*_2_ = 16, and *D* = 2	61.438 ± 1.383	64.41 ± 2.51
*F*_1_ = 8, *F*_2_ = 16, and *D* = 4	65.738 ± 1.572	72.27 ± 0.87
*F*_1_ = 16, *F*_2_ = 32, and *D* = 2	68.291 ± 0.524	72.67 ± 1.52
*F*_1_ = 16, *F*_2_ = 32, and *D* = 4	71.548 ± 0.631	76.58 ± 0.73
*F*_1_ = 3, *F*_2_ = 64, and *D* = 2	73.552 ± 1.263	76.45 ± 1.27
*F*_1_ = 3, *F*_2_ = 64, and *D* = 4	77.173 ± 0.703	79.87 ± 0.43
*F*_1_ = 6, *F*_2_ = 128, and *D* = 2	77.614 ± 0.399	80.50 ± 0.33
*F*_1_ = 6, *F*_2_ = 128, and *D* = 4	80.585 ± 0.728	82.66 ± 0.64

**Fig. 5:**
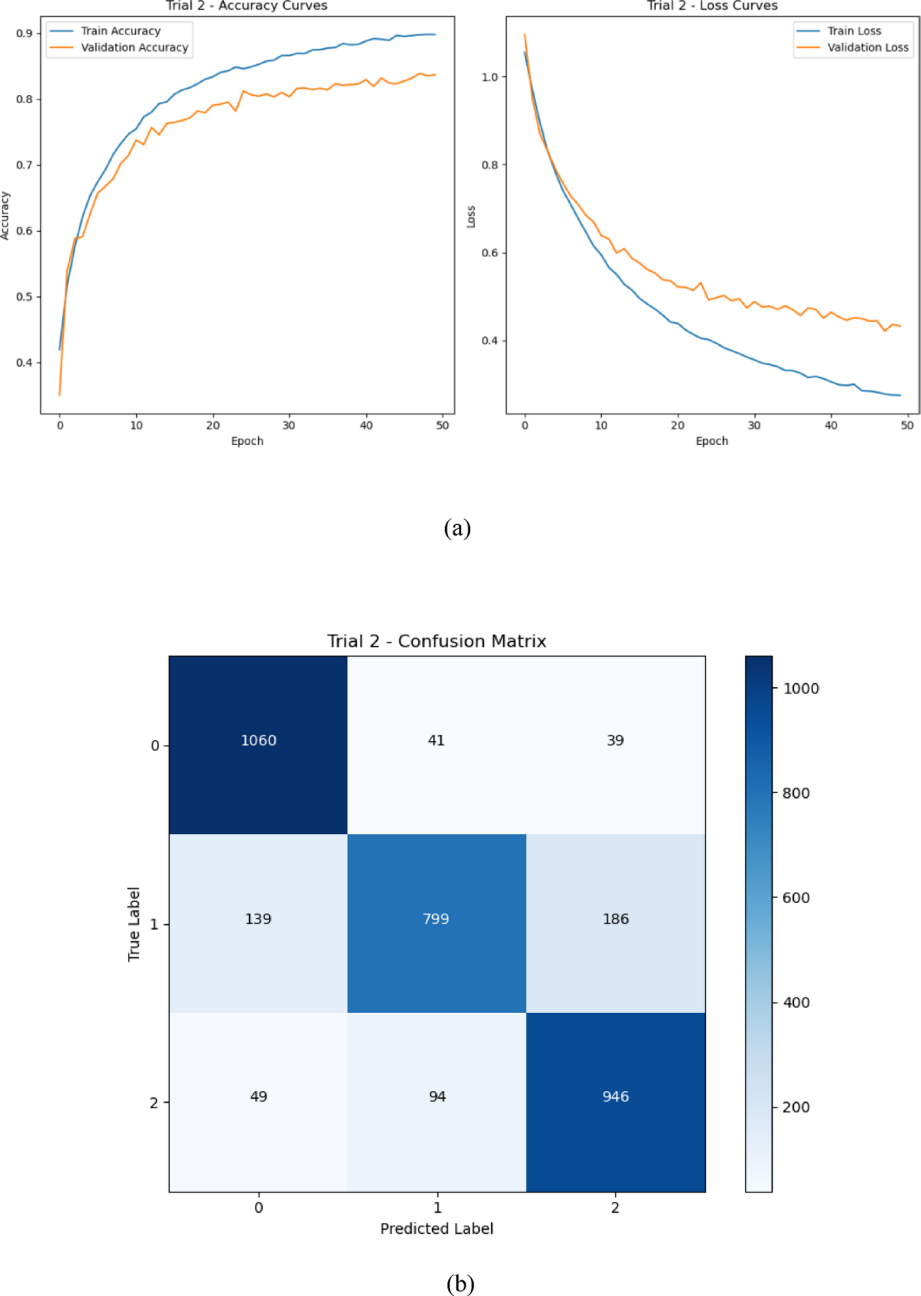
Performance metrics: (**a**) Accuracy and loss curve, (**b**) Confusion matrix.

From Table 23, it has been observed that using SMOTE continuously increases accuracy in all configurations, with higher filter values showing the most gains. When trained on the SMOTE-augmented data, the configuration (*F*_1_ = 6, *F*_2_ = 128, and *D* = 4) obtained the maximum ≈ 83% accuracy. The low value of standard deviation ranges from 0.33 to 2.51, indicating both strong and consistent performance. Figure 5a shows the accuracy and loss curve, and Fig. 5b shows the confusion matrix of the fifth trial for *F*_1_ = 6, *F*_2_ = 128, and *D* = 4 configuration with SMOTE. Accuracy and loss curves for all other configurations were not included to avoid redundancy, as their trends were similar but with inferior performance. The accuracy plots show that both training and validation accuracy gradually increase as the epochs progress, indicating the model is learning the patterns in the data. The loss keeps reducing over epochs for both training and validation sets, showing that the model is converging stably. The confusion matrix shows that most samples are correctly classified, as the diagonal values are higher.

### Statistical comparison of the SMOTE and the results without SMOTE results

To statistically evaluate the effect of SMOTE, a one-sided Wilcoxon test was used with the alternative hypothesis that the use of SMOTE leads to higher classification accuracy compared to training without SMOTE. For each configuration, the EEGNet model was trained for 50 epochs, and 5 independent trials were conducted with and without SMOTE. Since the accuracy results from the two conditions may not follow a normal distribution, a non-parametric Wilcoxon signed-rank test was preferred. Table 24 summarizes the *p*-value for all configurations. The results show that, for 8 out of 10 configurations, the one-sided *p*-value is less than the significance threshold α = 0.05, indicating a statistically significant performance improvement when SMOTE is used. The configuration *F*_1_ = 8*, F*_2_ = 16, and *D* = 2 and F_1 _= 32, F_2_ = 64, and D = 2 did not reach statistical significant (*p* > 0.05). The overall comparison across all configurations yielded a one-sided *p*-value (*p* < 0.001), confirming that SMOTE produces a significant improvement in EEG-based MWL classification accuracy.

**Table 24:** Statistical significance assessment using one-sided Wilcoxon signed-rank test.

Configuration	*p*-value	Significant (α = 0.05)
*F*_1_ = 4, *F*_2_ = 8, and *D* = 2	0.03125	TRUE
*F*_1_ = 4, *F*_2_ = 8, and *D* = 4	0.03125	TRUE
*F*_1_ = 8*, F*_2_ = 16, and *D* = 2	0.06250	FALSE
*F*_1_ = 8, *F*_2_ = 16, and *D* = 4	0.03125	TRUE
*F*_1_ = 16, *F*_2_ = 32, and *D* = 2	0.03125	TRUE
*F*_1_ = 16, *F*_2_ = 32, and *D* = 4	0.03125	TRUE
*F*_1_ = 3, *F*_2_ = 64, and *D* = 2	0.06250	FALSE
*F*_1_ = 3, *F*_2_ = 64, and *D* = 4	0.03125	TRUE
*F*_1_ = 6, *F*_2_ = 128, and *D* = 2	0.03125	TRUE
*F*_1_ = 6, *F*_2_ = 128, and *D* = 4	0.03125	TRUE

### SHAP analysis

In this study, a SHAP analysis was carried out to understand how the EEGNet model makes decisions. After training the model, we used the Gradient Explainer from the SHAP library, as the network is gradient-based. A subset of training data was used as background, and 50 samples from the test set were used to compute SHAP values to reduce computation while keeping the data representatives. SHAP values were calculated for every channel and time point. These values were then visualized using Global Feature Importance Plots, Local Explanation Summary, and Class-wise channel importance Plot.

#### Global feature importance plots

**Fig. 6:**
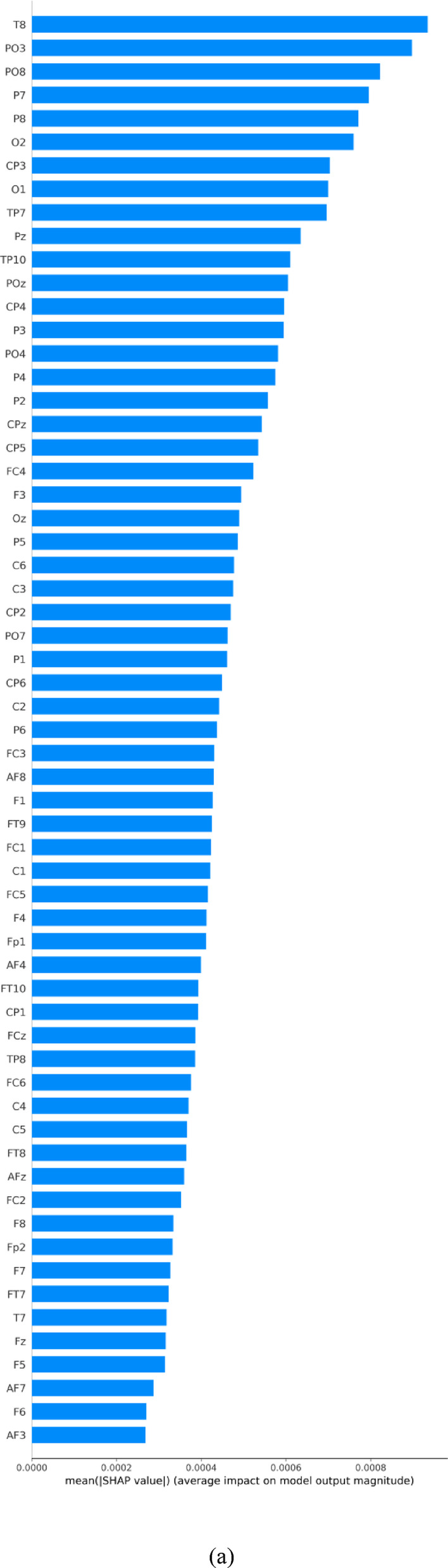

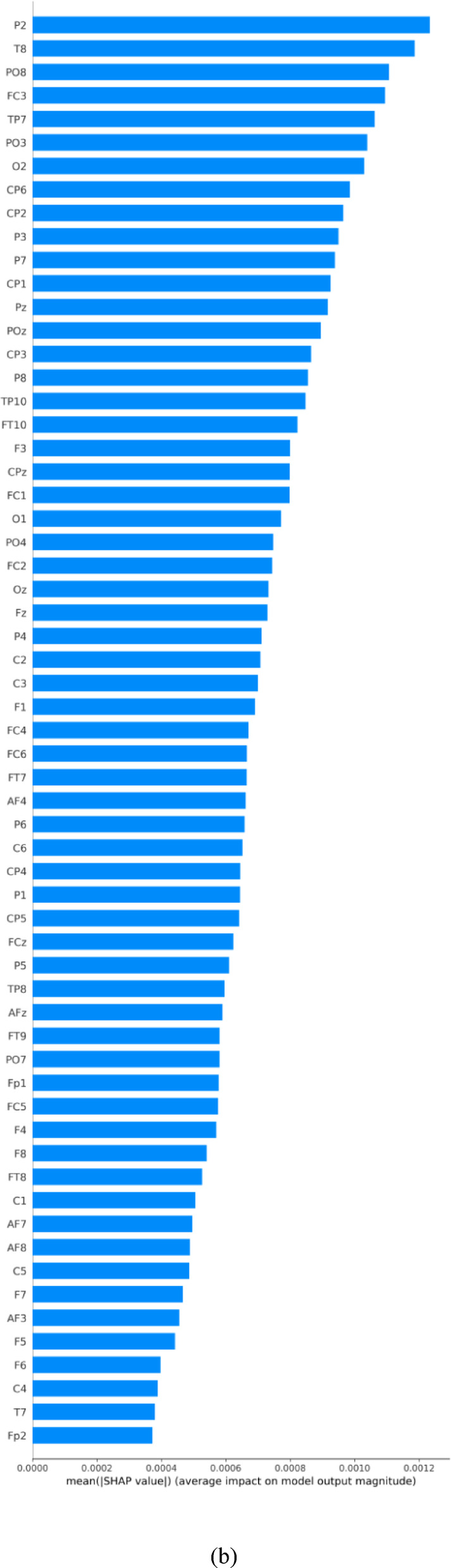
(**a**) Channel-wise global SHAP importance for EEGNet trained without SMOTE. (**b**) Channel-wise global SHAP importance for EEGNet trained with SMOTE.

Global feature importance plots for both, without SMOTE and with SMOTE, are shown in Fig. 6a and b, respectively. These plots revealed that the most informative EEG channels were largely consistent across both conditions, i.e., without and with SMOTE. In both situations, channels such as T8, PO3, PO8, O2, CP3, and Pz indicate higher SHAP values. These electrodes are located on parieto-occipital and temporal brain regions, which are associated with visuospatial processing, intentional control, and workload-related neural activity. We found that there is close correspondence between both conditions (without and with SMOTE) that indicates that SMOTE did not alter the underlying neurophysiological interpretation of the model; rather, the leading brain regions contributing to MWL discrimination remained the same in both training settings.

#### Local explanation summary

**Fig. 7:**
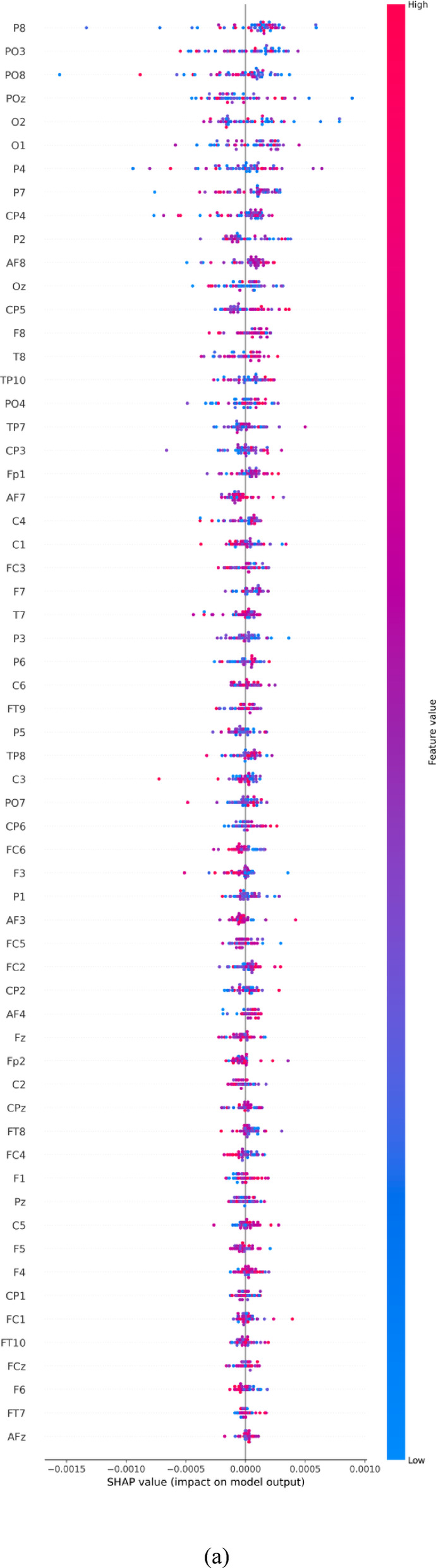

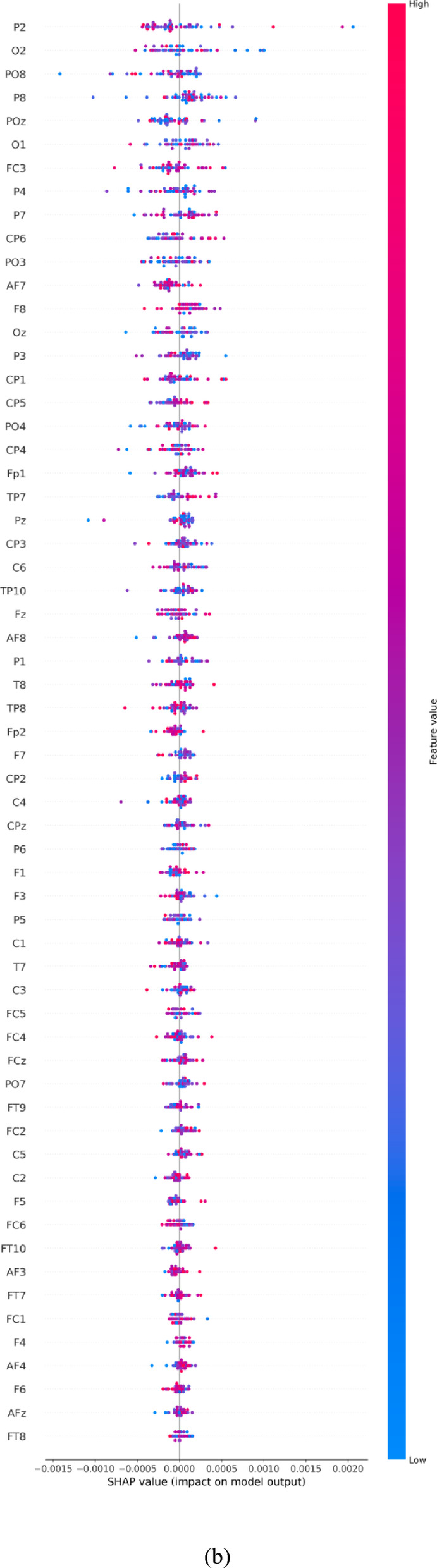
(**a**) Local (sample-level) SHAP value distribution for EEG channels (without SMOTE). (**b**) Local (sample-level) SHAP value distribution for EEG channels (with SMOTE).

Figure 7a and b, local explanation summary plots are shown for both cases without SMOTE and with SMOTE, which show the influence of features on model predictions at an instance level. Each dot in the plot represents one EEG sample. The horizontal axis represents the SHAP value; it provides information on whether the influence of that feature is linked to a favorable or unfavorable contribution to the prediction. A positive SHAP value indicates that an EEG feature increases the chance of a specific class, such as a high workload, and a negative value pushes the sample towards a different class. The red color indicates the high feature value, whereas the blue color indicates the low feature value. In both cases (without and with SMOTE), most of the influential variations are consistently observed over parieto-occipital and temporal electrodes (P8, PO3, PO8, O1, P7, and CP3). Although SMOTE increased the number of training samples, it did not affect dominant channels or the brain regions contributing to the model decisions. Instead, it resulted in a denser and smoother distribution of SHAP points, which means it did not change in neurophysiological interpretation.

#### Class-wise channel importance plot

**Fig. 8:**
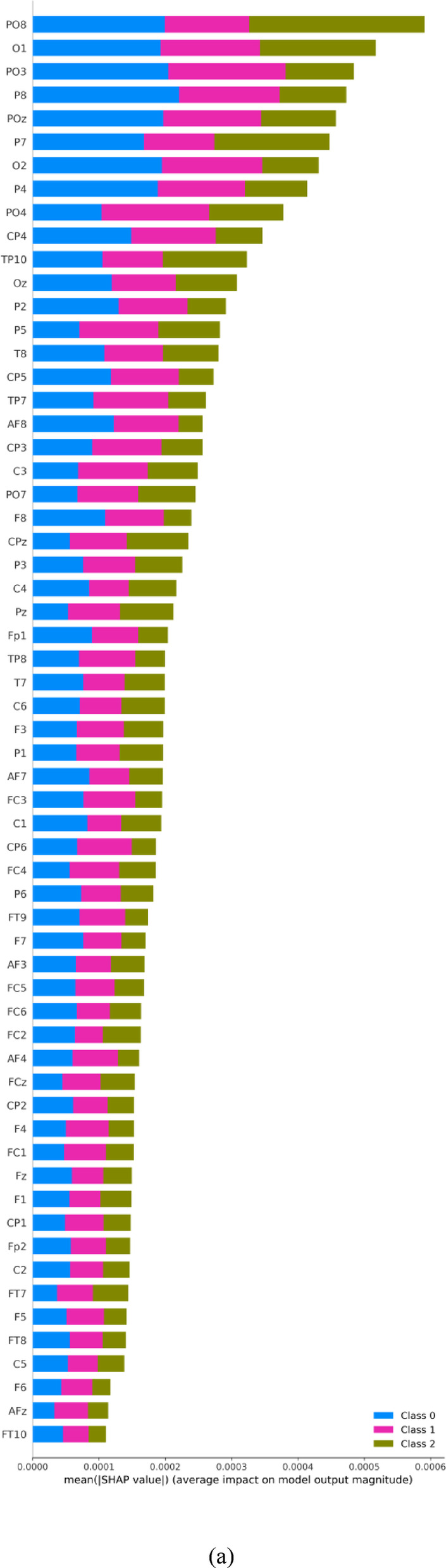

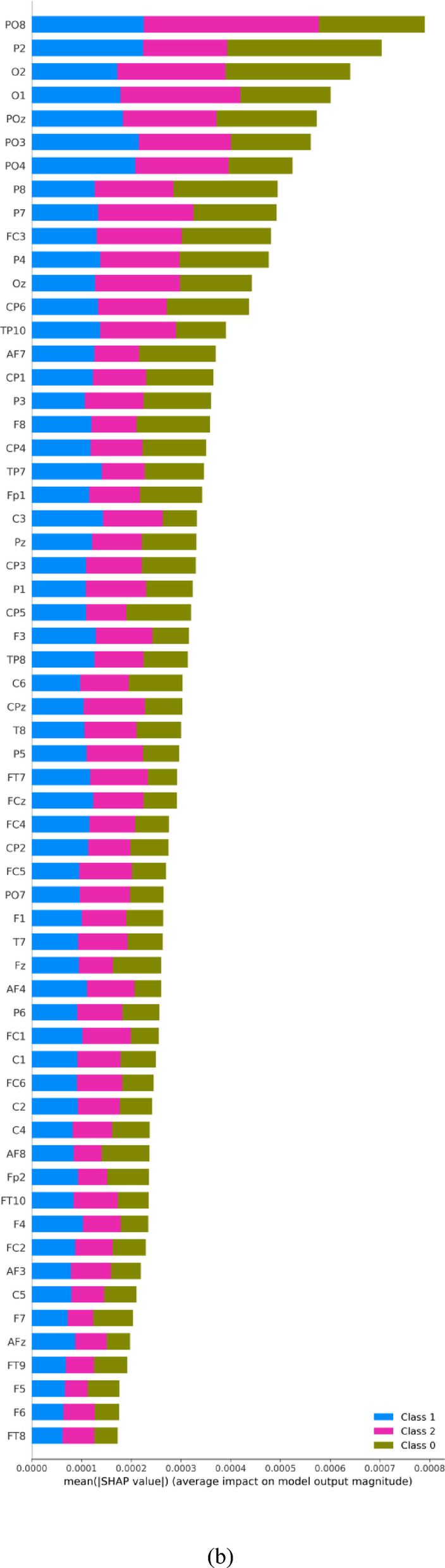
(**a**) Class-specific contribution of EEG channels to model output (without SMOTE). (**b**) Class-specific contribution of EEG channels to model output (with SMOTE).

Figure 8a and b shows the Class-wise channel importance Plot for both cases-without SMOTE and with SMOTE that compares the impact of different EEG channels on the classification of different classes (Low, Med, and High). The X-axis indicates the average magnitude of the SHAP value for the EEG channel; a high value means the feature has a greater impact on model prediction. On the other hand, the Y-axis indicates the electrode positions; the feature at the top makes a greater contribution to model prediction. The plot is broken down by class (blue for low workload or class 0, pink for medium workload or class 1, and green for high workload or class 2). From these plots, it has been observed that a bias toward the majority class is obvious in the first plot (without SMOTE), where the model mostly uses variables linked to Class 2 (green). Following the use of SMOTE (second plot), the feature contributions for each of the three classes become more evenly distributed, with pink (Class 1) and blue (Class 0) displaying greater influence, indicating that the model now takes into account all classes more equally. Both plots emphasize specific occipital and posterior channels, including PO8, O1, P8, P7, POz, O2, and CP4 as being crucial for classification. Overall, SMOTE increased sample density while preserving the dominant brain regions driving classification.

#### Quantitative analysis of channel importance using SHAP

To further investigate the quantitative analysis of the proposed model, SHAP analysis was performed on the trained EEGNet model. In this, for each subject and session, 10 samples were taken from each workload class, and SHAP values were computed. To check whether the important channel remained consistent across different subjects and sessions, we have calculated the mean and standard deviation of the SHAP values for each channel. The channels were then arranged according to their mean SHAP values, and the top ten channels that contribute most to the model’s predictions were summarized. As shown in Table 25, channels such as O1, P6, P7, POz, O2, etc., indicate the highest mean SHAP values. The small value of standard deviations indicates that these channels were consistent and their importance did not change across the subjects and sessions. This suggests that the model relies on a similar set of channels.

**Table 25:** Mean and standard deviation of SHAP values with channel ranking.

Rank	Channel	Mean SHAP values	Standard Deviation
1	O1	0.000135	0.000087
2	P6	0.000135	0.000089
3	P7	0.000122	0.000139
4	POz	0.000117	0.000058
5	O2	0.000117	0.000066
6	FT8	0.000108	0.000056
7	PO8	0.000107	0.000055
8	P8	0.000104	0.000072
9	CP5	0.000100	0.000054
10	PO4	0.000098	0.000049

Further, the SHAP values were analyzed separately for the three workload classes to examine channel’s contribution to individual workload class. Table 26 summarizes the mean SHAP value for three workload classes of the top ten important channels. From Table 26, it has been observed that all channels contribute to all classes with small variations. The low standard deviation values indicate that these channels remain stable and consistent across the workload classes.

**Table 26:** Comparison of channel-wise SHAP values across Easy, Medium, and High workload classes.

Channel	Easy class	Medium class	High class	Mean	Standard deviation
O1	**0.000165**	0.000123	0.000117	0.000135	0.000021
P6	0.000101	0.000140	**0.000162**	0.000135	0.000025
P7	**0.000149**	0.000107	0.000111	0.000122	0.000019
POz	0.000118	**0.000132**	0.000102	0.000117	0.000012
O2	0.000132	**0.000133**	0.000086	0.000117	0.000022
FT8	**0.000115**	0.000114	0.000094	0.000108	0.000010
PO8	0.000092	**0.000125**	0.000104	0.000107	0.000013
P8	0.000113	**0.000119**	0.000080	0.000104	0.000017
CP5	**0.000124**	0.000110	0.000066	0.000100	0.000025
PO4	0.000099	**0.000100**	0.000094	0.000098	0.000003

* Bold values shows maximum SHAP value among three workload classes.

From these observations, we found that mostly the important channels are located in parietal and occipital brain regions that are related to attention and visual processing required for the MWL task^32^. Furthermore, these results are also consistent with neuroscience findings, as these brain regions are known to be important for MWL processing.

## Discussion

This work has used the EEGNet model to classify MWL using EEG data. The model’s performance was shown to be consistently enhanced by increasing the number of temporal filters (F1), point-wise filters (F2), and the value of depth depth multiplier (D), according to experimental results. Higher filter settings improved classification performance by enabling EEGNet to extract more intricate and subtle spatiotemporal information from the EEG data. Additionally, the SMOTE technique is used to increase the number of samples, improving the model’s performance significantly. The model’s early bias toward the majority class was corrected as performance measures became more equal across all workload levels. This finding is also supported by SHAP-based interpretability analysis, which showed better sensitivity across all classes after SMOTE.

According to SHAP analysis, the parietal and occipital areas consistently have the most significant EEG channels, including PO3, PO8, P8, POz, P2, O1, O2, CP3, and Pz. These channels are located mainly over the parieto-occipital and centro-parietal regions of the brain, which are mostly responsible for attention, visual information processing, and integration of sensory inputs. MWL in our experiment involved continuous visual monitoring and information processing, which explains the stronger contribution from occipital and parieto-occipital regions (PO3, PO8, and O2), as these regions are closely related to visual cortex activity. The importance of channels CP3 and Pz reflects the involvement of the parietal cortex, which is associated with working memory,

decision making, and allocation of cognitive resources. Therefore, the channel relevance identified by SHAP is consistent with established evidence from neurophysiology studies.

The performance of the proposed approach was also compared with the state-of-the-art methods that have been applied to the same cross-session mental workload dataset, as shown in Table 27. Previous work reported moderate performance using various techniques. Spherical CNN with a rank-1 was used by Sedlar et al. and achieved 48.2% accuracy^26^, while Corsi et al. and Narayanan et al. used Riemannian geometry-based approaches and achieved 48.1% and 46.3% accuracy^27,28^, respectively. Botlton et al. and Sharma et al. used machine learning approaches and reported 44.6% and 38.4% accuracy^29,30^, respectively.

**Table 27:** Prior work on cross-session MWL dataset.

S. No	Authors	Method	Accuracy In (%)
1	Sedlar et al.^33^	Spherical CNN with rank-1 constraint	48.2
2	Corsi et al.^34^	Riemannian geometry + functional connectivity Features	48.1
3	Narayanan et al.^35^	Riemannian geometry	46.3
4	Bolton et al.^36^	Random Forest on classical features (300)	44.6
5	Sharma et al.^37^	Random Forest on CSP features	38.4
*6*	*Proposed work*	*EEGNet with SMOTE*	*≈ 83.0*

Our proposed approach, which combines EEGNet and SMOTE, outperforms all previous work on the same dataset and delivers greater classification accuracy (≈ 83%). These findings show how well deep learning and data augmentation can be combined to solve the data scarcity challenge, mainly in the context of MWL classifications.

## Conclusion

In this work, we demonstrate the classification of MWL using EEG data from multiple sessions with an EEGNet model. The optimal accuracy of ≈83% is attained for the configuration (*F*_1_ = 64, *F*_*2*_ = 128, and *D* = 4) by varying the main EEGNet parameters (depth multiplier *D*, temporal filter *F*_1_, and pointwise filters *F*_2_). Additionally, it has been noticed that SMOTE enhances performance across all scenarios. Our solution outperformed several state-of-the-art approaches, such as those based on spherical CNNs, Riemannian geometry, and classical machine learning algorithms, on the same cross-session MWL dataset. A consistent collection of informative electrodes that made a substantial contribution to the model’s predictions was also identified by the SHAP analysis, which provided insightful information about the most and least significant EEG channels. For training the model, data from all EEG channels were used, but only a subset of channels had a significant impact on classification choices, according to the SHAP analysis. In light of these results, in the future, we intend to investigate channel selection in subsequent research to simplify the model and maybe enhance classification performance by concentrating on the most pertinent EEG channels.

## Data Availability

The dataset used in this work is open access (online available) and can be found at: 10.5281/zenodo.5055046.

## Code availability

The code used in this work is publicly available at: https://github.com/213112003/EEG-Based-Mental-Workload-Classification.git.
